# Hospitals in the Arctic

**Published:** 1919-05-24

**Authors:** 


### HOSPITALS IN THE ARCTIC.

In a long article from its special correspondent at Archangel the *Times* has recently given (May 15) a most vivid and interesting account of the medical arrangements on the Kola Peninsula and elsewhere in the north of Russia. Partly owing to the wrecking of a ship which conveyed a large amount of hospital equipment the medical service in those regions has had to resort very largely to improvisation in the creation of hospitals, ambulance trains, and other forms of attendance for the wounded and sick. During the dark winter months, we are assured, there was but two or three hours of twilight in the twenty-four, and improvised hospitals had to be built by the light of flares in the Arctic cold, with sailors, engineers, and doctors as workmen. One such hospital now contains thirty-one buildings, a well-equipped operating theatre, an *x*-ray room, and a bacteriological laboratory—and a laundry is being built!

Another noteworthy feature of the medical side of this campaign has been the absence so far of typhus, typhoid, and scurvy, all three of which had been expected to cause trouble; in fact, with the exception of "a little" influenza, there has been no infectious disease. This should probably be read as a tribute to the sanitary and other preventive medical measures adopted by the R.A.M.C.; though the special correspondent suggests that part of the credit belongs to the men themselves. They are of a class (low category), he writes, that takes great care of itself, since the flush of youth is gone—the class that has begun to count the years that may remain, and "takes its medicine with profound seriousness." Whether the correspondent meant by this slightly ambiguous phrase the actually swallowing of physic, or the mental outlook of the soldier towards preventive medicine in general, is difficult to decide. Perhaps he used an elastic term to cover both! At least, it is satisfactory to learn that the troops there are comparatively free from serious sickness. In spite of their "low category" they ought to be, and doubtless are, more than a match for the Bolshevists, whose rates of sickness are almost certain to be enormous and ever-increasing. As a lesson in practical hygiene to the Russians, the Lapps, and other indigenous races, the medical arrangements of the Murmansk and Archangel forces ought to prove of the highest value, both immediately and as an object lesson for the future. The R.A.M.C. are, in fact, veritable medical missionaries in this respect. There is a section of opinion in this country which disapproves on political grounds of all these Russian expeditions, and would leave the Muscovite to stew or be stewed in the juices of Bolshevism. The political aspect of the matter is foreign to the purview of this Journal; but there is another side of the matter—namely, the hygienic, and from this aspect it is difficult to see anything but benefit arising from British intervention.

